# Levothyroxine Dosage Requirement During Pregnancy in Well-Controlled Hypothyroid Women: A Longitudinal Study

**DOI:** 10.5539/gjhs.v8n4p227

**Published:** 2015-08-31

**Authors:** Zahra Kashi, Adele Bahar, Ozra Akha, Samane Hassanzade, Leila Esmaeilisaraji, Zeinab Hamzehgardeshi

**Affiliations:** 1 Diabetes Research Center, Mazandaran University of Medical Sciences, Sari, Iran; 2 Mazandaran University of Medical Sciences, Sari, Iran; 3 Savadkook Health Center, Mazandaran University of Medical Sciences, Sari, Iran; 4 Department of Midwifery and Reproductive Health, Nasibeh Nursing and Midwifery Faculty, Mazandaran University of Medical Sciences, Sari, Iran; 5 Traditional and Complementary Medicine Research Centre, Mazandaran University of Medical Sciences, Sari, Iran

**Keywords:** hypothyroidism, levothyroxine, pregnancy

## Abstract

**Background:**

Untreated maternal hypothyroidism can have adverse effects on both the mother and fetus, but it can potentially be prevented by adequate levothyroxine replacement. This study was conducted to determine what percentage of hypothyroid pregnant women who were taking levothyroxine needed to adjust their medication dosage, and when and how much it should be increased.

**Methods:**

In this longitudinal study, 81 well-controlled hypothyroid women (TSH≤ 2.5 mIU/L) were monitored throughout pregnancy. Thyroid function tests were performed before conception, after the first missed menstrual period, in the second and third trimesters of pregnancy and one month after delivery. Levothyroxine dosage was adjusted according to TSH levels measured.

**Results:**

Of the 81 pregnancies studied, the pregnancy outcomes were 74 full-term births, six abortions and one pre-term birth. The levothyroxine dosage needed to be increased in 84% (CI95%= 74-90) of the pregnancies (OR=5.2, CI95%= 2.9-9.4). Most levothyroxine dose adjustments were made in the first trimester of gestation. The levothyroxine requirement increased 50% (CI95%= 41-59) in the first trimester, 55% (CI95%= 45-64) in the second trimester and 62% (CI95%= 52-72) in the third trimester. Levothyroxine dosage was decreased for 6 cases (7.4%), and no adjustment was made for 7 women (8.6%).

**Conclusions:**

Increases in levothyroxine dosage administered in pregnancy appear to be indispensible in the majority of patients with well-controlled hypothyroidism, especially in the first trimester. However, this change was not universal and levothyroxine dosage decreased in a few cases and remained unchanged in others.

## 1. Background

Thyroid dysfunction in pregnant women can affect both mother and her fetus throughout pregnancy. This condition is associated with higher prevalence of abortions, still births, maternal anemia, pregnancy-induced hypertension, postpartum hemorrhage, preterm labor, fetal distress, low birth weight, and neuropsychological impairment (Hallengren, Lantz, Andreasson, & Grennert, 2009; Karaca & Akpak, 2015; LaFranchi, Haddow, & Hollowell, 2005; Wasserstrum & Anania, 1995). However, it has been documented that if maternal hypothyroidism is managed appropriately, it will have no side effect on maternal or developing fetus throughout the pregnancy (Tan, Cheng, & Caughey, 2006; L. Yassa, E. Marqusee, R. Fawcett, & E. K. Alexander, 2010).

In addition to several reports regarding the requirement for adjustment of levothyroxine dose in hypothyroid pregnant women, there are various controversies surrounding the amount and timing of this dosage adjustment. Different studies stated that an increase in the levothyroxine dose is necessary in 50-85% of pregnancies to maintain the preconception serum TSH level (Abalovich et al., 2002; Alexander et al., 2004; Kaplan, 1996; Mandel, Larsen, Seely, & Brent, 1990) and the levothyroxine dose need to be increased 23-50% compared to the pre-pregnancy dosage and after parturition it could be decreased in most women (Alexander et al., 2004; Hallengren et al., 2009; Kaplan, 1992; Mandel, 2004; Mandel et al., 1990; Pekonen et al., 1984). It is recommended that hypothyroid women can increase levothyroxine dose by approximately 30% as pregnancy is confirmed (Alexander et al., 2004).

There are several possible explanations for an increased need for T4 during pregnancy including increment of absorption and distribution of thyroxine due to gravid uterus and mass of fetal placental unit and increase in serum concentration of thyroxine-binding globulin (TBG) that is a consequence of an estrogen-induced increase in the glycosylation of TBG and leads to decreased hepatic clearance of this protein (Alexander et al., 2004; Mandel, 2004; Mandel et al., 1990). Conversely, there are other studies that rejected this increased need for levothyroxine requirement during pregnancy and stated that the cause of this increment in other studies may be due to impaired absorption of thyroxine (T4) possibly related to ingestion of exogenous agents such as iron, calcium, and vitamins (Chopra & Baber, 2003; Girling & de Swiet, 1992). Most of previous studies were retrospective and in some studies participants were patients with thyroid cancer who needed higher levothyroxine dose.

This study was designed in order to assess that what percentage of thyroxine-treated pregnant women with well-controlled hypothyroidism needed to increase their drug dosage during pregnancy according to thyroid function tests (TFTs) and when and how much the drug should be increased.

## 2. Methods

In this longitudinal study (2007-2010), 81 women with hypothyroidism who were planning to become pregnant and were referred to the university endocrine outpatient clinics in Sari, Iran were enrolled. Inclusion criteria were age range of 15-45 years, confirmed hypothyroid state before the pregnancy, and no history of thyroid malignancy, thyroid surgery or radioactive iodine therapy.

The cases were chosen among eligible pregnant hypothyroid women referred to using simple random sampling method. After controlling the hypothyroidism in subjects (i.e, TSH level≤ 2.5 mIU/L), the subjects were allowed to be pregnant.

After confirming pregnancy, blood sample was obtained and based on the result of laboratory the levothyroxine dose was adjusted (according to the Kaplan guidelines) (11):

**Table:** 

TSH level(mIU/L)	Adjustments made in levothyroxine dosage
TSH ≤ 2.5	None
2.5<TSH≤5	25 µg increase
5<TSH≤10	50 µg increase
10<TSH≤20	75 µg increase
TSH>20	100 µg increase

This test was repeated after a month and after achieving the acceptable TSH (≤ 2.5 mIU/L), thyroid tests were repeated in each trimester and one month after parturition in order to adjusting levothyroxine dosage. In all laboratory tests, at least 6 hours after using T4, 5 cc blood was taken and electrochemiluminescence method was used.

In addition, for all subjects a questionnaire consisting of data about age, weight, duration of hypothyroidism, hypothyroidism type, consumption of supplements including iron and calcium, levothyroxine dose before conception, family history of hypothyroidism, history of abortion and parity were filled.

By inhibiting the absorption of levothyroxine administered concurrently with vitamins and iron enriched agents, the subjects were asked to take these components at least 4 hours after ingesting T_4_. The levothyroxine tablet administered for by all subjects was product of Iran Hormone Drug Co (Tehran, Iran).

The ethics committee of Mazandaran University of Medical Sciences approved the protocol of the study. All participants were informed of the purpose and methods of the research.

### 2.1 Statistics

Mean (±SD, standard deviation) along frequencies and them 95%CI, Odd ratio and its 95%CI were reported. All analyses were done by the SPSS software. P values ≤ 0.05 were considered statistically significant.

## 3. Results

In this study 81 hypothyroid women who were planning to become pregnant with mean (SD) age of 26.9 (±4.9) years were followed. The average body mass index (BMI) was 35.4 (±3.8) kg/m^2^. About 56% of the subjects tested positive for anti-thyroid peroxidase antibody (anti-TPO). The hypothyroidism condition was clinical in 54.3% (44 women) and subclinical in 45.7% (37 women) of the cases. The first degree family history of thyroid dysfunction was positive in 39.5% of the sample. Fifty-five women (67.9%) were nulliparous and 16 (19.8%), 9 (9.9%), and 2 women (2.5%) were experiencing their second, third and fourth pregnancies, respectively. Twenty-one percent (17 women) had once, 2.5% (2 women) had twice and 1.2% (one woman) had three times of abortion history in the past.

Blood samples were taken 5 times from subjects to measure hormone levels and levothyroxine dosage was adjusted accordingly (Table 1). Six patients aborted and one had preterm labor.

**Table 1:** Mean (SD) levothyroxin dosages and TSH levels before, during and after pregnancy

	Preconception	First trimester	Second trimester	Third trimester	After parturition
TSH mIU/L	1.33±0.78	3.35±2.73	2.64±1.72	2.28±1.27	1.29±1.79
Levothyroxine dose (µg/day)	87.4±38.9	107.3±43.5	113.9±18.2	121.4±40.8	108.7±44.4

In 6 subjects (7.4%), the administered levothyroxine dosage was decreased and in 7 subjects (8.6%), no change in dosage was required. An increase in the levothyroxine dose was necessary in 84% (CI95%= 74-90) of pregnancies (odd= 5.2, CI95%= 2.9-9.4). In the first trimester, 59.3% of the subjects needed increase in their levothyroxine dosage. This percentage increased to 72.8% in the second trimester and finally until delivery 84% of the cases need an increase in their levothyroxine dosage in comparison with preconception dosages (Table 2). The levothyroxine dosage required was increased fifty percent (CI95%= 41-59) in the first trimester, 55% (CI95%= 45-64) in the second trimester, and 62% (CI95%=52-72) in the third trimester. In subjects with positive anti-TPO, 92.3% needed increased medication dosage and in those with negative anti-TPO, 70% needed such adjustment (P= 0.04).

**Table 2:** Frequency (percentage) of the pregnant women whose levothyroxine dosage was adjusted (increased, decreased, or remained unchanged) at different stages of pregnancy

Gestational age	Levothyroxine requirement dosage compared to preconception		

	Unchanged N (%)	Decreased N (%)	Increased N (%)
First trimester	48 (59.3)	5 (6.2)	28 (34.6)
Second trimester	59 (72.8)	6 (7.4)	10 (12.3)
Total	68 (84.0)	6 (7.4)	7 (8.6)
After parturition	53 (65.4)	8 (9.9)	15 (18.5)

Enhanced requirement for T4 was not significantly different between subjects with clinical and those with subclinical hypothyroidism (84.1% vs. 83.8%, P= 0.7).

Participants who needed an increased levothyroxine dose were compared to those whose dosage was decreased or remained unchanged. The level of anti-TPO antibody and the duration of hypothyroidism before gestation were significantly different between the two groups (P= 0.0001 and P= 0.001); (Table 3).

**Table 3:** Comparison of the studied variables between the pregnant women who needed increased levothyroxine dosage during pregnancy and those who did not

	Needed levothyroxine increase	No need to increase levothyroxine	P value
Age(year)	26.7 (±4.8)	27.85 (±5.5)	0.4
preconception TSH (mIU/L)	1.35 (±0.79)	1.21 (±0.78)	0.5
BMI (kg/m^2^)	26.3 (±3.85)	26.3 (±3.47)	0.9
Anti-TPO antibody level (IU/mL)	270.50 (±288.10)	35.67 (±56.24)	0.0001
Delta weight change during pregnancy, Kg	10.34 (±11.4)	10.36 (±6.0)	0.3
Preconception weight, (Kg)	67.75 (±10.2)	71.50 (±11.1)	0.9
Preconception levothyroxine dosage, (µg/day)	88.4 (±40.9)	81.9 (±26.7)	0.6

## 4. Discussion

Based on the results obtained here, 84% of the pregnant hypothyroid women studied needed an increase in their levothyroxine dose. Most subjects who required such adjustment were detected in the first trimester of pregnancy. Moreover, the average dose increments in each trimester were 50%, 55%, 62%, respectively. These results agree with other studies that suggested the levothyroxine dose should be increased during pregnancy (Alexander et al., 2004; Hallengren et al., 2009; Kaplan, 1992; Mandel, 2004; Mandel et al., 1990; Pekonen et al., 1984). The previous study showed that increase in L-T4 at pregnancy significantly reduces the risk of maternal hypothyroidism during the first trimester (Leila Yassa, Ellen Marqusee, Rachael Fawcett, & Erik K Alexander, 2010). Patients with positive anti-TPO antibody test results had higher requirement of levothyroxine during pregnancy in comparison to those who tested negative for anti-TPO antibody. Our results are in agreement with Negro et al. (2006) and Poppe and Glinoer (2003) reports, who noted that pregnant women with positive anti-TPO antibody results experienced higher rate of obstetrical complications and required higher doses of levothyroxine replacement. Another study suggested that women whose dose is being adjusted preconception period need more likely to increasing of dose (Kothari & Girling, 2008).

Two questions are raised by these results: first, why does this increase occur? Second, what should be our approach to hypothyroidism in pregnancy?

There are some possible explanations for this increased requirement for levothyroxine during pregnancy. The increased level of TBG, increase in plasma volume and the influence of the mass of the fetal-placental unit are some explanations for this increased requirement (Ain, Mori, & Refetoff, 1987; Alexander et al., 2004). Increases in serum concentration of TBG is the result of estrogen-induced increment in the glycosylation of TBG and declined hepatic clearance of this protein (Ain et al., 1987). Thyroxine metabolism by the fetal placental unit also can be a possible cause of an incremented need for levothyroxine in the later phases of gestation and to the decline in the need after parturition (Huang, 2005). To maintain adequate concentrations of thyroid hormone during pregnancy, T4 and triiodothyronine (T3) production are increased physiologically by the thyroid gland throughout a normal pregnancy but this compensation cannot happen in women with hypothyroidism. In healthy women, serum concentrations of total T3 and T4 increase during the first trimester and reach a plateau either late in the second trimester or early in the third trimester (Guillaume, Schussler, & Goldman, 1985; Weeke et al., 1982).

The temporary chorionic gonadotropin-induced increase of the thyroxine production rate in normal gestation can provide largely this increment (Mandel et al., 1990). Because thyroid glands in hypothyroid women are not able to respond either to thyrotropin or to chorionic gonadotropin, the incremented requirement for thyroxine is not achieved and the serum thyrotropin concentration increases. Our results that these women required higher dose of levothyroxine throughout gestation, rather than transiently, also suggest that there is a constant increase in thyroxine production throughout pregnancy. The other interpretation is that these women have a constant acceleration in thyroxine metabolism (Mandel et al., 1990).

Another issue of interest is how should be our approach to these cases; in hypothyroid pregnant women who were on thyroxine treatment before conception, most of them (in our study 84%) needed increased levothyroxine dosage (Alexander et al., 2004; Chopra & Baber, 2003; Hallengren et al., 2009; Idris, Srinivasan, Simm, & Page, 2005; Kaplan, 1992; Mandel, 2004; Mandel et al., 1990; McDougall & Maclin, 1995) although a few studies reported that fewer subjects (21-38%) needed such adjustment(Girling & de Swiet, 1992; Pekonen et al., 1984). The disagreement of these studies with our results can be due to uncontrolled hypothyroidism before the pregnancy and also use of different TSH levels as the goal of treatment in other studies (Alexander et al., 2004; Hallengren et al., 2009; Idris et al., 2005).

The need to adjust levothyroxine dose manifests itself as early as at 4-8 weeks of gestation, therefore justifying the adjustment of levothyroxine replacement to ensure that maternal euthyroidism is maintained during early gestation. There are two views regarding this issue, while some thyroidologist recommend to predict the expected increase in serum TSH by increment in levothyroxine dose before conception in order to reach preconception TSH concentration in the low-normal range (0.3-0.7 mU/L) which minimizes the number of hypothyroid women in the first post conception visit (Rotondi et al., 2004). Other authors suggest increasing the levothyroxine dose by approximately 30% as soon as pregnancy is confirmed (Alexander et al., 2004). However, by accessing to performing TSH measurement, we can decide about this increment according to the biochemical results and not a blind approach. It should be considered that some hypothyroid women are able to keep a normal serum TSH level in the first trimester of pregnancy and some of those who maintain a normal serum TSH concentration until the second trimester may still need to increment in levothyroxine dose during late gestation to maintain euthyroid status (Mandel, 2004).

In summary, serum free T_4_ and TSH level should be measured within 1 month after start of treatment. The overall purpose is acquiring and keeping normal free T_4_ and TSH values throughout pregnancy and for achieving this, levothyroxine treatment should be titrated to achieve a serum TSH value of less than 2.5 mIU/L (Negro et al., 2006). As normal thyroid function tests are achieved after treatment, they should be rechecked every 6-8 weeks. In cases that TFTs remain abnormal, we should adjust levothyroxine dosage and repeat TFTs every 30 days until normalization of tests.

The limitation of our study was not measuring free T4 in all subjects. This serum marker is useful for hypothyroidism recognition during pregnancy. Here, the diagnosis of hypothyroidism was confirmed before the pregnancy and according to present guidelines the drug adjustment in pregnancy is done according to TSH level (Stagnaro-Green et al., 2011).

## 5. Conclusions

According to the present study, most of well-controlled hypothyroid pregnant women needed increased dosage of thyroid hormone after pregnancy. The levothyroxine dosage was increased 50% in the first trimester and then needed to be added 5% every trimester thereafter. As some pregnant women did not need such adjustment and in some cases even the levothyroxine dosage was decreased, we recommend that the drug adjustment should be according to laboratory results if accessible.
